# Lymphomatoid Granulomatosis with Splenomegaly and Pancytopenia

**DOI:** 10.3779/j.issn.1009-3419.2010.01.17

**Published:** 2010-01-20

**Authors:** Abolhasan HALVANI, Mohammad Bagher OWLIA, Ramin SAMI

**Affiliations:** 1 Department of Internal Medicine, Shaheed Sadoughi hospital, Shaheed Sadoughi University of Medical Sciences, Safaeieh, Yazd, Iran; 2 Shaheed Sadoughi Hospital, Shaheed Sadoughi University of Medical Sciences, Safaeieh, Yazd, Iran; 3 Shaheed Beheshty university, Tehran, Iran

**Keywords:** Lymphomatoid granulomatosis, Pancytopenia, Splenomegaly

## Abstract

Lymphomatoid granulomatosis (LG) is an angiocentric lymphoproliferative disease. It usually involves lung, skin, and central nervous system, but splenomegaly and pancytopenia are the rare manifestations of the disease. We report a 15-year-old boy presented with fever, dry cough and dyspnea from two months ago, after admission patient had nodular lesions on the left leg and hepatosplenomegaly. Then he manifested neurologic signs such as seizure, aphasia and right-sided hemiplegia. Chest X-ray and CT scan revealed bilateral pulmonary nodules predominantly in lower lobes and peripheral lung fields. Laboratory exams showed pancytopenia. Skin biopsy was done, and histopathological examination and immunohistochemistry evaluation confirmed lymphomatoid granulomatosis. He was treated with steroid and cyclophosphamide but succumbed by neurologic involvement.

## Introduction

Liebow *et al*^[1]^ first described lymphomatoid granulomatosis (LG) as a triad of polymorphic lymphoid infiltrate, angiitis, and granulomatosis in 1972. It is a rare Epstein-bar virus associated with systemic angiodestructive lymphoproliferative disease, currently it is described as an Epstein-Barr-viruspositive and T-cell-rich B-cell lymphoproliferative disorder^[2]^. Eventhough LG mimics Wegener's granulomatosis and frequently discussed in the context of the vasculitides, it is a lymphoproliferative disorder, not primary vasculitis^[3]^. The disease generally occurs in patients in their 40s and has a strong male predominance^[4, 5]^.

Patients often presents with fever, malaise, cough and dyspnea, the presenting symptom and signs are suggestive of infectious disease^[6]^.

We present a 15-year-old man who was admitted because of cough, dyspnea, hepatosplenomegaly, pancytopenia, nodular and alveolar lung lesions and demonstrated many difficulties in diagnosis of lymphomatoid granulomatosis.

## Case description

A 15-year-old boy was admitted with a 2-month history of fever, decreased appetite and nonproductive cough. Cough and fever progressed despite of antibiotics prescription for presumed community-acquired pneumonia a few weeks prior to admission.

At the time of admission, he had fever, tachycardia, respiratory distress and splenomegaly. In laboratory findings, he had pancytopenia; urine analysis and ESR was normal, blood and urine culture was negative. Gram stain of his sputum showed gram positive cocci. Acid fast bacillus was not found in sputum smear. HBsAg and Anti-HCV and Anti-HIV were negative. ANA, Anti ds-DNA, p-ANCA and c-ANCA were requested but all of them were negative. Peripheral blood smear and bone marrow aspiration were normal. Echocardiography also was normal. His chest X-ray and CT-scan of thorax showed bilateral and peripheral nodular and alveolar pattern, predominantly in lower lobes of the lung. Mediastinal lymphadenopathy and pleural effusion were not seen (Fig 1). Abdominal sonography and CT showed hepatosplenomegaly. Splenomegaly was more prominent than hepatomegaly. Para-aortic lymphadenopathy was not seen. Bronchoscopy and open lung biopsy were not performed because the patient's guardian did not permit. Two days later, we saw a few small brown nodular lesions on the left leg (Fig 2). Skin excisional biopsy was taken. One day later, he developed right hemiplegia, seizure and aphasia. Brain CT scan showed a broad hypo dense area on the left temporal and basal ganglia that had little enhancement after injection of Ⅳ contrast (Fig 3). At this time, the result of skin biopsy was prepared. There was severe infiltration of lymphocytes and histiocytes and atypical lymphocytes on the subcutaneous and deep parts of the derma. The main aggregation of the cells was around the vessels and nerves and appendices of the skin. The endothelial layer of the vessels had inflammation, but there was no morphologic pattern of vasculitis. Lymphoid cells had positive reaction with CD45RO: so lymphomatoid granulomatosis (LG) was diagnosed (Fig 4). He was treated with steroid pulse therapy and oral cyclophosphamide but after 12 days he expired without any clinical improvement and any response to the treatment.

**1:**
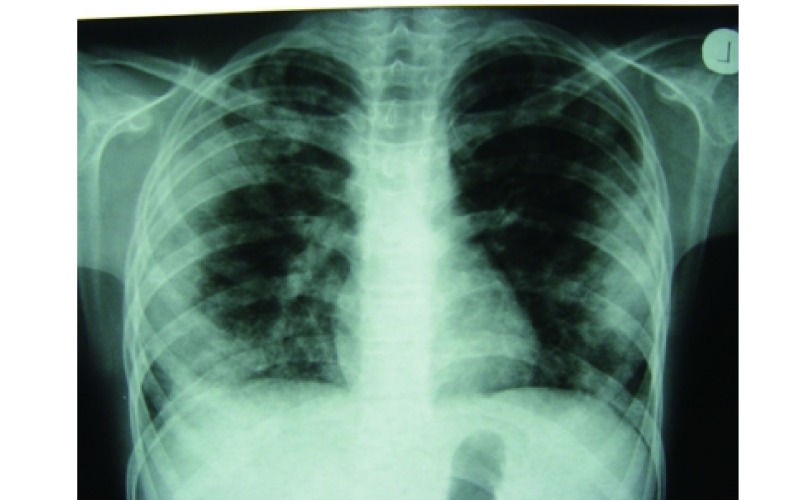
Chest X-ray and CT-scan of thor

**2:**
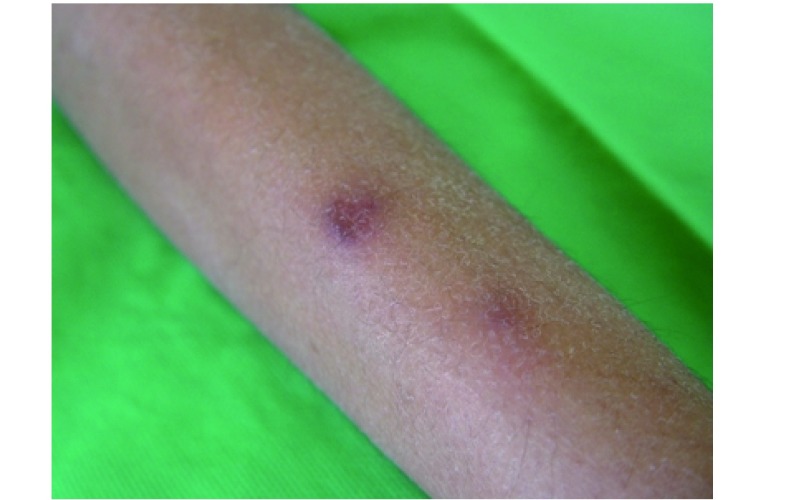
Few small brown nodular lesions on the left leg

**3:**
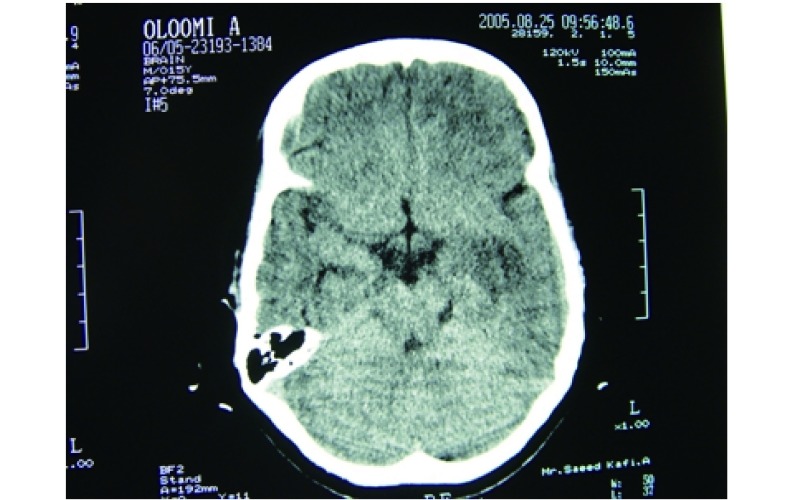
Brain CT scan

**4:**
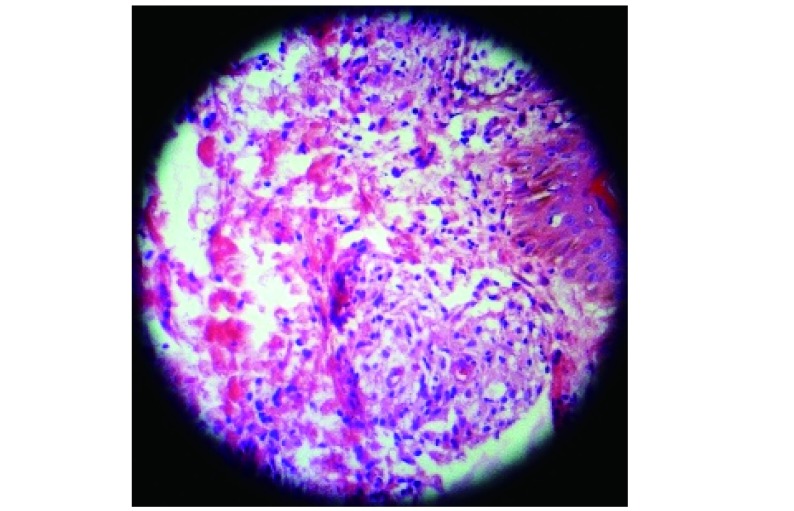
Lymphomatoid granulomatosis

## Discussion

Lymphomatoid granulomatosis can affect any organ but it is found most frequently in the lung, central nervous system and skin. Pulmonary involvement is almost invariably a feature of LG. The radiological appearance of LG is multiple bilateral pulmonary nodules involving the lower and peripheral lung fields. Cavitations, atelectasis or lobar obstruction, large masslike disease, and pneumothorax may be seen in LG. The pulmonary lesions typically wax and wane and some areas may regress while others are progressing^[4, 6]^. Sometime, lymphomatoid granulomatosis shows CT features such as peribronchovascular distribution of nodules and coarse irregular opacities, small thin-walled cysts, and small conglomerating nodules^[7]^. The reported patient had nodular and alveolar lesions mostly in the peripheral and lower lobes.

Skin is the second most commonly affected organ in LG that has been reported in up to 55% of cases. Skin can reveal rash, subcutaneous nodules, or ulceration. These lesions are generally painless, but can occasionally be tender and pruritic. They are usually small and nonconfluent and often on the extremities^[6]^. The reported patient had painless nodular skin lesions on the left leg a few days after the onset of his illness.

The CNS is involved in 20% of LG patients but it is rare as an initial presentation^[8]^. It is characterized by focal deficits in an asymmetric distribution, sometimes with seizures, ataxia, cranial nerve disorders, and peripheral neuropathy^[6, 8]^. The CT appearance of cerebral involvement is diverse. The disease which is most commonly seen as low-attenuation areas in the white matter is sometimes hemorrhagic and often enhances with Ⅳ contrast material^[9]^. Neurologic manifestation is an adverse prognostic factor. In our patient, the CT revealed nearly extensive hypo dense lesions at the left temporal area and basal ganglia.

Renal and hepatic involvement is unusual. Hepatic involvement may carry a worse prognosis. Lymph node and splenic involvement is rare and do not appear to adversely affect prognosis^[6]^. Our patient had prominent hepatosplenomegaly.

There are no characteristic laboratory values in LG^[6]^. CBC is usually normal but sometimes reveals mild leucopenia or leukocytosis^[1]^. This patient had pancytopenia that initially improved by treatment.

The diagnosis of LG requires examination of affected tissue biopsy, usually lung, skin, or head and neck^[3]^. The biopsies from lung, skin or CNS has the same value^[1]^. There is a necrotizing, angiocentric, and angiodestructive infiltrative process. The key microscopic picture is the presence of poly-morphic infiltration of plasma cells, immunoblasts, and typical large lymphoid cells with a tendency to involve the wall of pulmonary vessels and to collect in the subendothelial spaces. The multinucleated giant cells and necrotizing changes of Wegener's granulomatosis are not present. Prominent vascular involvement without necrotic changes is also seen in the cutaneous lesions of LG. The necrotic component is prominent and results in extensive parenchymal destruction. Real granulomatose inflammation, however, does not occur^[1, 10]^. Immunohistochemistry shows that most of the cells-small to medium-sized lymphocytes are T cells (CD45RO+); however, a much smaller population of medium-sized to large atypical cells are B cells (CD20+). In each case, these cells are combined. Most cases of LG involving in the lung represent a proliferation of Epstein-Barr virus infected B-cells with a prominent T-cell reaction and vasculitis, distinguishing these cases from angiocentric " T-cell lymphomas" in other sites (such as the head and neck)^[11]^. We planed open lung biopsy as diagnostic procedure for this patient but because the patient's guardian did not permit and the biopsies from lung and skin has the same value, we performed skin excisional biopsy. Histopathology and immunohistochemistry evaluation confirmed lymphomatoid granulomatosis.

The disease usually progresses relentlessly, eventuating in death. However, spontaneous remissions have been described. Its pathophysiology is unclear in numerous respects, thus making it difficult to diagnose and treat^[12]^. In earlier studies responses are noted to regimen of oral cyclophosphamide and corticosteroid (prednisolone)^[2, 13]^. Responses to INF-alpha 2b are reported anecdotally, but the experiences are limited to only few cases^[3, 14]^. There are reports of both clinical and pathological remission by anti-CD20 monoclonal antibody (Rituximab)^[15]^. Clinicopathologic studies have shown that progression of LG to lymphoma occurs in 12%-47% of patients, and the mortality rate has been reported to be between 53% and 63.5%^[7]^. Adverse prognostic factors include neurologic manifestations, and large numbers of atypical lymphoreticular cells within the pulmonary infiltrate. Unilateral chest lesions and large numbers of small lymphocytes and histiocytes within the infiltrate are associated with a better prognosis^[13]^.

Prognosis is generally poor. The death usually occurs within 14 months from the time of diagnosis. Half or more of patients succumb to the disease within five years^[5]^.

This patient was treated with steroid pulse therapy and oral cyclophosphamide but after 12 days he expired without any clinical benefit of the treatment.
